# Generating fragrant oilseed rape using CRISPR/Cas9-mediated gene editing

**DOI:** 10.1093/plphys/kiae660

**Published:** 2024-12-23

**Authors:** Jian Wu, Jingyi Xu, Ancheng He, Sichao Ren, Yi Ye, Wenjing Lei, Yu Liu, Xia Hua, Chunjie Wei, Li Lin, Hui Zhang, Youping Wang

**Affiliations:** Key Laboratory of Plant Functional Genomics of the Ministry of Education, Yangzhou University, Yangzhou 225009, China; Key Laboratory of Plant Functional Genomics of the Ministry of Education, Yangzhou University, Yangzhou 225009, China; Key Laboratory of Plant Functional Genomics of the Ministry of Education, Yangzhou University, Yangzhou 225009, China; Key Laboratory of Plant Functional Genomics of the Ministry of Education, Yangzhou University, Yangzhou 225009, China; Key Laboratory of Plant Functional Genomics of the Ministry of Education, Yangzhou University, Yangzhou 225009, China; Key Laboratory of Plant Functional Genomics of the Ministry of Education, Yangzhou University, Yangzhou 225009, China; Key Laboratory of Plant Functional Genomics of the Ministry of Education, Yangzhou University, Yangzhou 225009, China; Key Laboratory of Plant Functional Genomics of the Ministry of Education, Yangzhou University, Yangzhou 225009, China; College of Life Science, Shanghai Normal University, Shanghai 200234, China; Key Laboratory of Plant Functional Genomics of the Ministry of Education, Yangzhou University, Yangzhou 225009, China; College of Life Science, Shanghai Normal University, Shanghai 200234, China; Key Laboratory of Plant Functional Genomics of the Ministry of Education, Yangzhou University, Yangzhou 225009, China; Jiangsu Key Laboratory of Crop Genomics and Molecular Breeding, Yangzhou University, Yangzhou 225009, China

## Abstract

Knocking out 2 functionally redundant aldehyde dehydrogenase-encoding genes via gene editing leads to 2-acetyl-1-pyrroline accumulation in oilseed rape, which can be used to develop fragrant lines.

Dear Editor,

Oilseed rape (OSR, *Brassica napus* L.) is the third-largest oilseed crop worldwide and the largest in China. OSR is also an exceptional source of early green fodder, haylage, and high-protein cake/meal. Interestingly, OSR also emerged as an ornamental crop owing to its beautiful seasonal flowers, with flower tourism contributing up to $1.57 million in daily revenue in China (Xiao et al. 2021). China proposed the development of multifunctional utilization of OSR oil, flower, vegetable, honey, feed, and fertilizer capabilities (Xiao et al. 2022).

Fragrances heavily influence the value of agricultural products. For instance, fragrance is a key determinant of rice (*Oryza sativa* L.) quality and market price. Rice fragrance is primarily attributed to 2-acetyl-1-pyrroline (2AP), resulting from the disruption of the *betaine aldehyde dehydrogenase 2* (*BADH2*) gene in family 10 of the aldehyde dehydrogenase (ALDH10) superfamily (Chen et al. 2008). The inhibition of BADH2 blocks the conversion of γ-aminobutyraldehyde (GABald) into γ-aminobutyric acid (GABA), leading to an accumulation of 2AP and fragrance emission. Fragrant varieties resulting from mutations in the *BADH2* include, but are not limited to, rice (Chen et al. 2008; Tang et al. 2021; Hui et al. 2022), maize (Wang et al. 2021), sorghum (Zhang et al. 2022), millet (Zhang et al. 2023), and soybean (Juwattanasomran et al. 2011; Qian et al. 2022). It was unclear whether the function of *BADH2* was conserved in *Brassica* and whether variations in OSR *BAHD2* homologs would result in fragrant germplasm.

In *Arabidopsis thaliana* there are 2 *BADH* genes, *ALDH10A8* and *ALDH10A9*. Two homologs of each gene were found in OSR, namely *BnaA07.ALDH10A8*, *BnaC06.ALDH10A8*, *BnaA06.ALDH10A9*, and *BnaC03.ALDH10A9*, respectively (Fig. 1A). These genes exhibit a high amino acid sequence similarity with their homologs in *Arabidopsis* (>93%, Supplementary Fig. S1), implying functional conservation. Both *Arabidopsis* ALDH10A8 and ALDH10A9 exhibit catalytic activity, converting GABald into GABA in vitro (Zarei et al. 2016; Jacques et al. 2020). Studies in *Arabidopsis* revealed that silique length is reduced in *aldh10a8 aldh10a9* double mutants, and 80% of the seeds within these siliques fail to develop properly. Defects were not observed in *aldh10a8* and *aldh10a9* single mutants (Jacques et al. 2020). Therefore, we proposed knocking out only one of the *ALDH10A8* or *ALDH10A9* genes.

**Figure 1.**
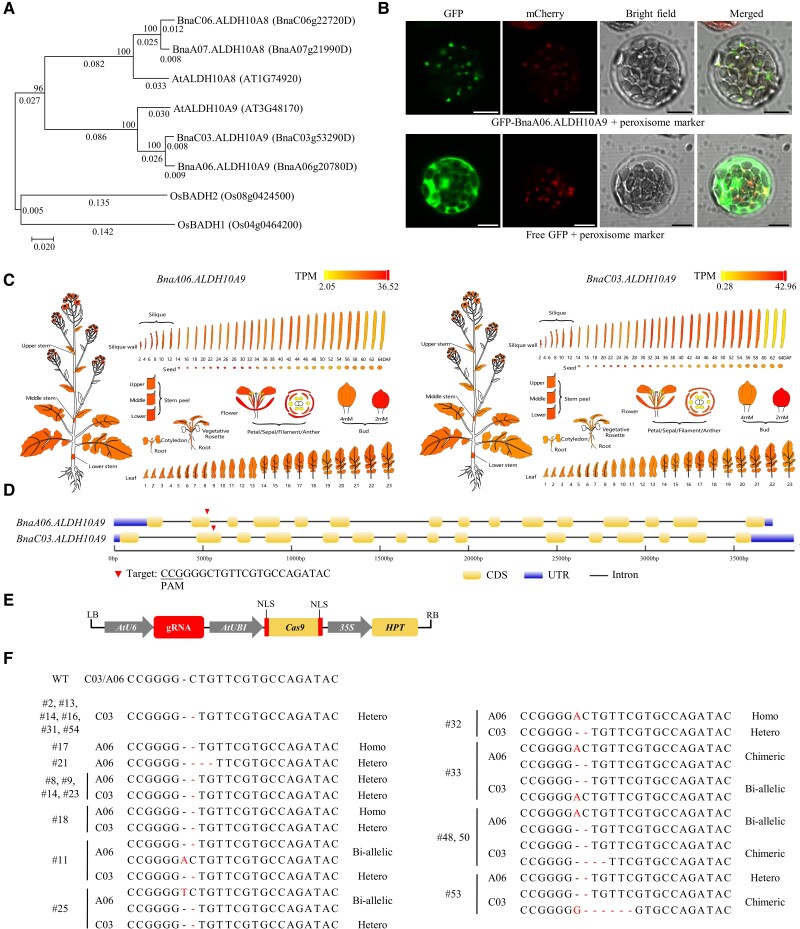
Generation of *Bnaaldh10a9* mutants in OSR by CRISPR/Cas9-mediated gene editing. **A)** Neighbor-joining tree shows the phylogenetic relationships of BADH2 proteins in *Bna*, *At,* and *Os*. Bootstrap values (1,000 replicates) are shown at each branch as a percentage. A branch length scale bar indicates the number of amino acid residue substitutions per site. The phylogenetic tree was constructed using MEGA7 software. **B)** Subcellular localization of the GFP-BnaA06.ALDH10A9 fusion proteins in *Arabidopsis* protoplasts. Bars = 10 μm. mCherry fused to the peroxisome localization signal serine-lysine-leucine (mCherry-SKL) was used as a peroxisome marker. **C)** The electronic fluorescent pictograph viewer displays the tissue expression patterns of *BnaALDH10A9* based on the BnTIR transcriptomic database (Liu et al. 2021). TPM, transcripts per million mapped reads. DAF, days after flowering **D)** Gene structure and the target site of the CRISPR/Cas9 vector. The protospacer-adjacent motif (PAM) is underlined. **E)** Schematic diagram of the CRISPR/Cas9 vector. gRNA, guide RNA; NLS, nuclear location signal; HPT, hygromycin phosphotransferase. **F)** Sequences at the target site in the T_0_ generation according to TA cloning and Sanger sequencing. Dashes represent deletions; red letters represent insertions. WT, wild type; Hetero, heterozygous; Homo, homozygous; A06, *BnaA06.ALDH10A9*; C03, *BnaC03.ALDH10A9*. Chimeric, containing 1 WT and 2 different mutations simultaneously.

As in rice, ALDH10A9 proteins in *Arabidopsis* and OSR carry the serine-lysine-leucine C-terminal peroxisomal targeting signal, whereas ALDH10A8 proteins do not (Supplementary Fig. S1). A subcellular localization experiment confirmed that BnaA06.ALDH10A9 specifically localizes to the peroxisome (Fig. 1B). Transcriptomic analysis revealed that both *BnaA06.ALDH10A9* and *BnaC03.ALDH10A9* are consistently expressed at all growth stages and tissue types (Fig. 1C), indicating that these 2 *BnaALDH10A9* homologs may exhibit functional redundancy. Therefore, we attempted to generate fragrant OSR by knocking out both *BnaALDH10A9* homologs using CRISPR/Cas9 genome editing.

We designed one guide RNA (gRNA) that specifically targeted the second exon of both *BnaALDH10A9* genes (Fig. 1D). The CRISPR/Cas9 construct contained the gRNA driven by the Arabidopsis U6 snoRNA promoter (*AtU6*) promoter, a Cas9 gene driven by the *AtUBI* promoter and a hygromycin phosphotransferase selection marker (Fig. 1E). This construct was transformed into the wild type (WT) OSR line J9712 through *Agrobacterium*-mediated transformation, resulting in 46 independent positive transformants in the T_0_ generation. The target genomic regions were amplified by PCR, followed by TA cloning and Sanger sequencing. We identified 20 T_0_ plants (43.5%) with editing events at the target site, including 8 single and 12 double mutants (Fig. 1F). Among the mutations examined, 62.5% of loci were heterozygous mutations, 25% were homozygous or bi-allelic, and 12.5% were chimeric mutations (Fig. 1F). The majority were 1 bp insertions or deletions (Fig. 1F). No editing events occurred at both *BnaALDH10A8* genes.

Three mutant lines were further examined: line #17 (homozygous single mutation in *BnaA06.ALDH10A9*), line #2 (heterozygous single mutation in *BnaC03.ALDH10A9*), and line #18 (double mutant, homozygous mutation in *BnaA06.ALDH10A9* and heterozygous mutant in *BnaC03.ALDH10A9*) (Fig. 1F). All 3 were single cytosine nucleotide deletions leading to translational frame shifts (Fig. 1F). Following self-pollination of these mutants, homozygous single and double mutants were identified from the T_1_ progeny using allele-specific PCR markers for *BnaA06.ALDH10A9* and *BnaC03.ALDH10A9*, respectively (Fig. 2A; Supplementary Fig. S2 and Table S1).

**Figure 2.**
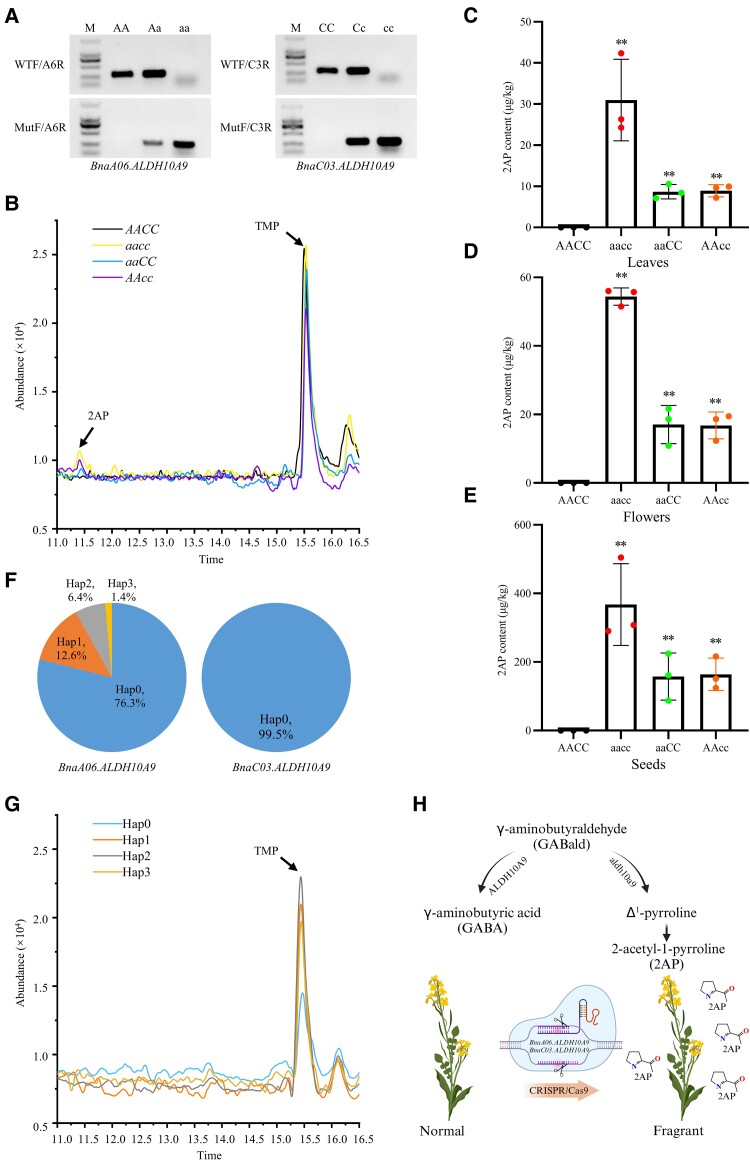
*Bnaaldh10a9* mutants accumulate 2AP. **A)** Allele-specific markers were developed to discriminate the WT, heterozygous, and homozygous mutants for *BnaA06.ALDH10A9* (AA, Aa, and aa, respectively) and *BnaC03.ALDH10A9* (CC, Cc, and cc, respectively). WTF and MutF were the allele-specific primer; A6R and C3R were the copy-specific primer. **B)** Total ion chromatograms of 2AP and 2,4,6-TMP (TMP, as an internal standard) in leaves of the *Bnaaldh10a9* mutants and the WT (J9712). **C–E)** 2AP content in leaves (C), flowers (D) and dried mature seeds (E). Data are shown as the mean ± SD (*n* = 3). Statistical significance was determined by a two-tailed Student’s *t*-test. **, *P* < 0.01. In (B–E), AACC, J9712; aacc, *bnaa06.aldh10a9 bnac03.aldh10a9* double mutant; aaCC, *bnaa06.aldh10a9* single mutant; AAcc, *bnac03.aldh10a9* single mutant. **F)** Haplotype analysis of *BnaALDH10A9* based on the BnIR database (Yang et al. 2023). The frequency of each haplotype is shown. **G)** Total ion chromatograms of 2AP and TMP in seeds of the accession from each haplotype of *BnaA06.ALDH10A9*. **H)** Schematic diagram showing the generation of fragrant OSR by CRISPR/Cas9. Created in BioRender (https://BioRender.com).

Next, we verified fragrance in the homozygous lines of the T_2_ generations. Interestingly, our organoleptic assessments revealed that the *bnaa06.aldh10a9 bnac03.aldh10a9* homozygous double mutant flowers emitted a jasmine-like fragrance, unlike the WT flowers, which lacked this fragrance. To quantify this fragrance, the 2AP contents in leaves, flowers, and dried mature seeds were measured by gas chromatography-mass spectrometry. A 2AP peak was observed at about 11.4 min in all tissues from the *bnaaldh10a9* single and double mutants, but not in the WT (Fig. 2B; Supplementary Figs. S3 to S4). In all tissues examined, the 2AP content was similar in both single mutants, but was 2.3 to 3.5 times higher in the double mutant (Fig. 2C to E). For the double mutant, the highest content was observed in the mature seeds (367.6 μg/kg), followed by the flowers (54.4 μg/kg) then the leaves (31.0 μg/kg) (Fig. 2C to E). Specifically, in mature seeds, the 2AP content in the double mutant is comparable to levels found in maize and sorghum (Wang et al. 2021; Zhang et al. 2022). These findings indicated that both *BnaALDH10A9* homologs are involved in the biosynthesis of 2AP in OSR, exhibiting functional redundancy.

*Arabidopsis aldh10a9* mutants are more sensitive to salinity than WT (Zarei et al. 2016); however, our observations suggest that the *bnaa06.aldh10a9 bnac03.aldh10a9* double mutant may not exhibit increased sensitivity (Supplementary Figs. S5 to S6). The key agronomic traits of the double mutant were not significantly altered compared with the WT (Supplementary Table S2).

We further investigated whether natural variation in *BnaALDH10A9* genes results in fragrant germplasm. We conducted haplotype analysis using the BnIR database (Yang et al. 2023). Among 2,311 OSR accessions, there were 4 haplotypes for *BnaA06.ALDH10A9*, and only 1 haplotype for *BnaC03.ALDH10A9* (Fig. 2F). We randomly selected 5 accessions from each haplotype of *BnaA06.ALDH10A9*, and found that none accumulated 2AP in seeds (Fig. 2G). These findings indicated that the observed natural variations in *BnaA06.ALDH10A9* do not lead to the loss of gene function and concurrent 2AP accumulation.

In conclusion, our findings demonstrate that the disruption of BnaALDH10A9 leads to the accumulation of 2AP in OSR (Fig. 2H), as in cereal crops like rice, maize, sorghum, and millet. Naturally fragrant OSR germplasms are lacking. We used CRISPR/Cas9-mediated editing to knockout *BnaALDH10A9* homologs, resulting in fragrant OSR lines that hold potential for enhancing the multifunctional utilization of OSR, particularly in terms of its oil, flower, vegetable, and feed capabilities.

## Supplementary Material

kiae660_Supplementary_Data

## Data Availability

No new data were generated or analysed in support of this research.

## Supplementary data

The following materials are available in the online version of this article.

**Supplementary Table S1.** Sequences of the primers used in this study.

**Supplementary Table S2.** Agronomic traits of WT and *bnaa06.aldh10a9 bnac03.aldh10a9* plants.

**Supplementary Figure S1.** The amino acid sequence alignment of BADH2 proteins in *Brassica napus* (*Bna*), *Arabidopsis thaliana* (*At*) and *Oryza sativa* (*Os*).

**Supplementary Figure S2.** Schematic representation of the allele-specific markers developed to distinguish between WT, heterozygous mutants and homozygous mutants (Mut) for *BnA06.ALDH10A9* and *BnC03.ALDH10A9*.

**Supplementary Figure S3.** Total ion chromatograms of the 2AP authentic standard and 2,4,6-trimethyl pyridine (TMP, as an internal standard).

**Supplementary Figure S4.** Total ion chromatograms of 2AP and 2,4,6-TMP (TMP, as an internal standard) in flowers and seeds of the *Bnaaldh10a9* mutants and WT (J9712).

**Supplementary Figure S5.** Salt stress tolerance of the *bnaa06.aldh10a9 bnac03.aldh10a9* double mutant (aacc) and WT.

**Supplementary Figure S6.** Root growth of the *bnaa06.aldh10a9 bnac03.aldh10a9* double mutant (aacc) and WT (J9712) under varying salt concentrations.

## Dive Curated Terms

The following phenotypic, genotypic, and functional terms are of significance to the work described in this paper:

ALDH10A8 Gramene: AT1G74920
ALDH10A8 Araport: AT1G74920

ALDH10A9 Gramene: AT3G48170
ALDH10A9 Araport: AT3G48170

TMP CHEBI: CHEBI:77120
