# Supplementary material for: Generating fragrant oilseed rape using CRISPR/Cas9-mediated gene editing
Source: Plant Physiol. 2024 Dec 23;197(1):kiae660. doi: 10.1093/plphys/kiae660 (PMC11702980; doi:10.1093/plphys/kiae660)
Supplement: kiae660_Supplementary_Data [file kiae660_supplementary_data.pdf]

Supplementary data

Table S1. Sequences of the primers used in this study.

Primer name	Sequence (5'-3')	Usage
GFP-ALDH10A9-F	tggatgaactatacaaagagATGGCGATTACGGTGCCGC	Construction of subcellular localization vector
GFP-ALDH10A9-R	gatcggggaaattcgagTTAAAGCTTGGAAGGAGGTTTG	
sgRNA-ALDH10A9-F	gtaaGTATCTGGCACGAACAGCCC	Construction of CRISPR/Cas9 vector
sgRNA-ALDH10A9-R	aaacGGGCTGTTCGTGCCAGATAC	
WTF	TGGTCTAAAGCATCCGGGGCT	Allele-specific marker for *BnaA06.ALDH10A9* (AA)
ALDH10A9-A6R	GTTACAAGCAACCAGGGTCCA	
MutF	TGGTCTAAAGCATCCGGGGTG	Allele-specific marker for *bnaa06.aldh10a9* (aa)
ALDH10A9-A6R	GTTACAAGCAACCAGGGTCCA	
WTF	TGGTCTAAAGCATCCGGGGCT	Allele-specific marker for *BnaC03.ALDH10A9* (CC)
ALDH10A9-C3R	GCAAGGGTTACAAGCAACCAGGTAT	
MutF	TGGTCTAAAGCATCCGGGGTG	Allele-specific marker for *bnac03.aldh10a9* (cc)
ALDH10A9-C3R	GCAAGGGTTACAAGCAACCAGGTAT	
U6F	CCCAGGATTAGAATGATTAGGC	Identification of positive transgenic plants
sgRNA-A9-R	GGGCTGTTCGTGCCAGATAC	
ALDH10A9-A6A3F	CCTTCGCCGTCAAACACT	PCR amplification of target fragment of *BnaA06.ALDH10A9*
ALDH10A9-A6R	GTTACAAGCAACCAGGGTCCA	
ALDH10A9-A6A3F	CCTTCGCCGTCAAACACT	PCR amplification of target fragment of *BnaC03.ALDH10A9*
ALDH10A9-C3R	GCAAGGGTTACAAGCAACCAGGTAT	

Supplementary Table S2. Agronomic traits of wild type and *bnaa06.aldh10a9 bnac03.aldh10a9* plants.

Line	Plant height (cm)	Branch initiation height (cm)	First effective branch number	Silique number per plant	Silique lengh (cm)	Silique seed number	Thousand-seed weight (g)	Oil content (%)
J9712	125.75 ± 7.23	16.50 ± 4.36	8.67 ± 0.58	443.67 ± 83.48	7.90 ± 0.47	24.62 ± 2.64	4.17 ± 0.21	39.17 ± 0.83
Double mutant	129.25 ± 9.64	20.25 ± 6.95	9.50 ± 2.38	381.50 ± 37.48	8.36 ± 0.80	23.62 ± 1.82	4.03 ± 0.16	38.80 ± 1.70

Note: Values are means ± SD. A total of 15 plants were randomly selected for each line.

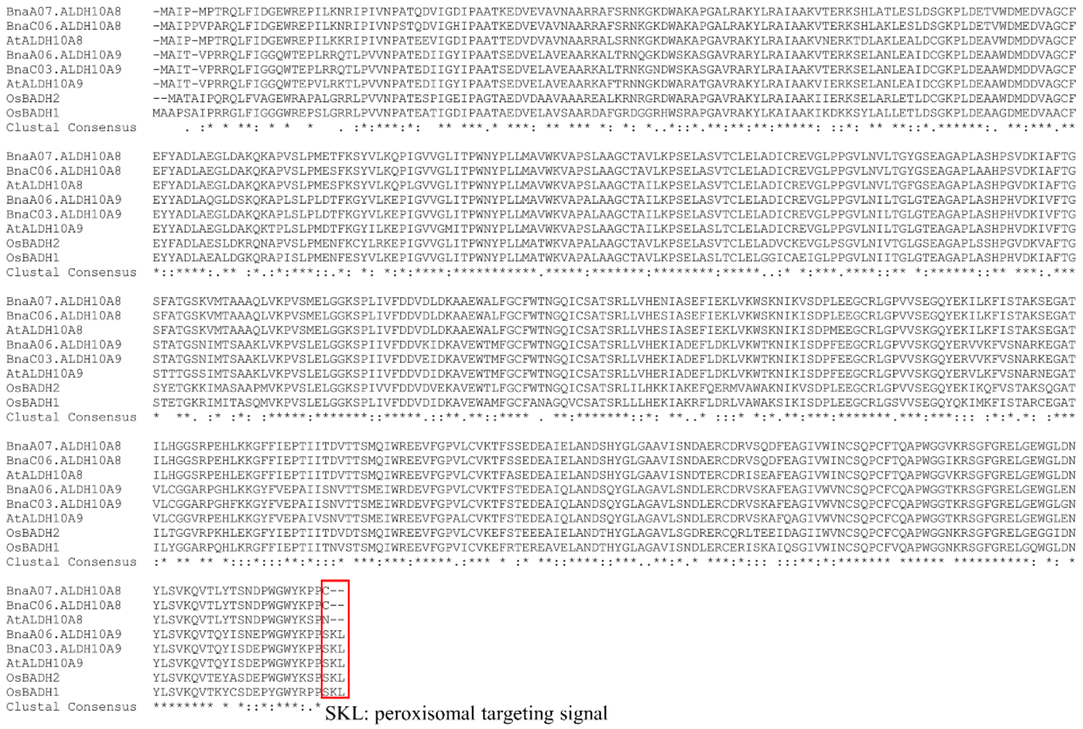

Supplementary Figure S1. The amino acid sequence alignment of betaine aldehyde dehydrogenase 2 (BADH2) proteins in *Brassica napus* (*Bna*), *Arabidopsis thaliana* (*At*) and *Oryza sativa* (*Os*). Multiple amino acid sequence alignments were conducted with ClustalX (v2.1, http://www.clustal.org/clustal2/) and viewed by the software BioEdit (v7.0.9.0, https://bioedit.software.informer.com/). The symbols *, :, and . indicate completely conserved, strongly conserved, and weakly conserved residues, respectively, across all sequences in the alignment.

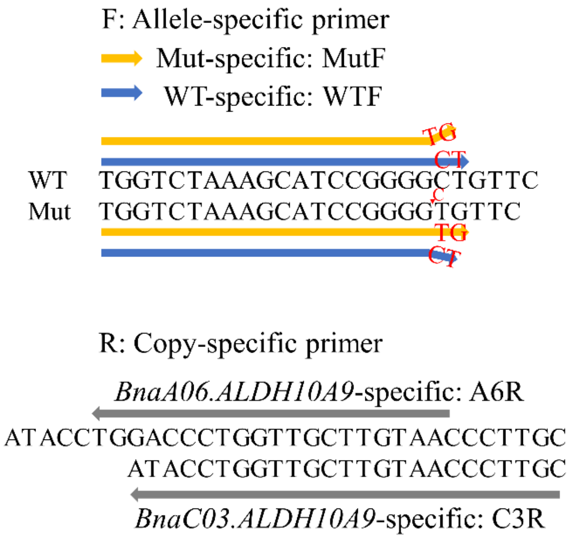

Supplementary Figure S2 Schematic representation of the allele-specific markers developed to distinguish between wild type (WT), heterozygous mutants and homozygous mutants (Mut) for *BnA06.ALDH10A9* and *BnC03.ALDH10A9*. The triangular symbol followed by the letter "C" indicates a missing base "C," while red letters in the primer highlight specific bases unique to different primers.

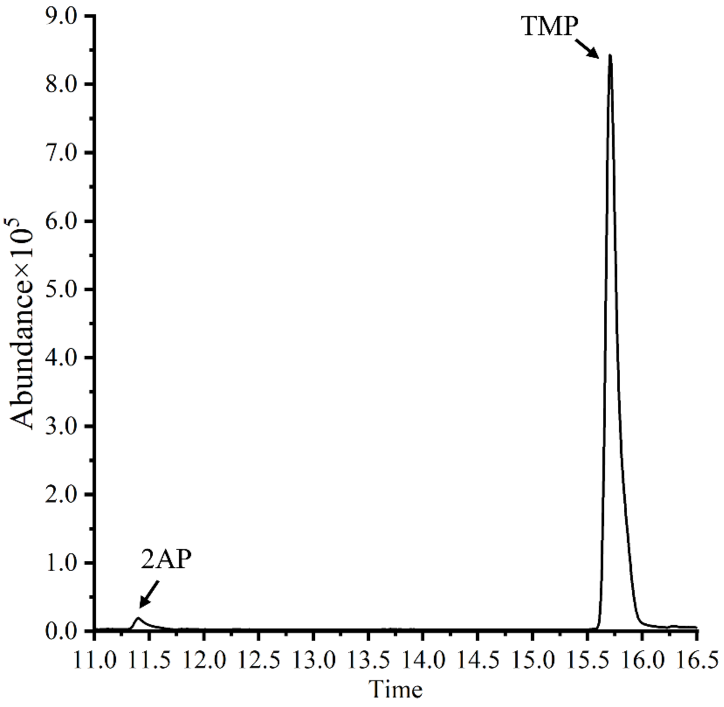

Supplementary Figure S3 Total ion chromatograms of the 2-acetyl-1-pyrroline (2AP) authentic standard and 2,4,6-trimethyl pyridine (TMP, as an internal standard).

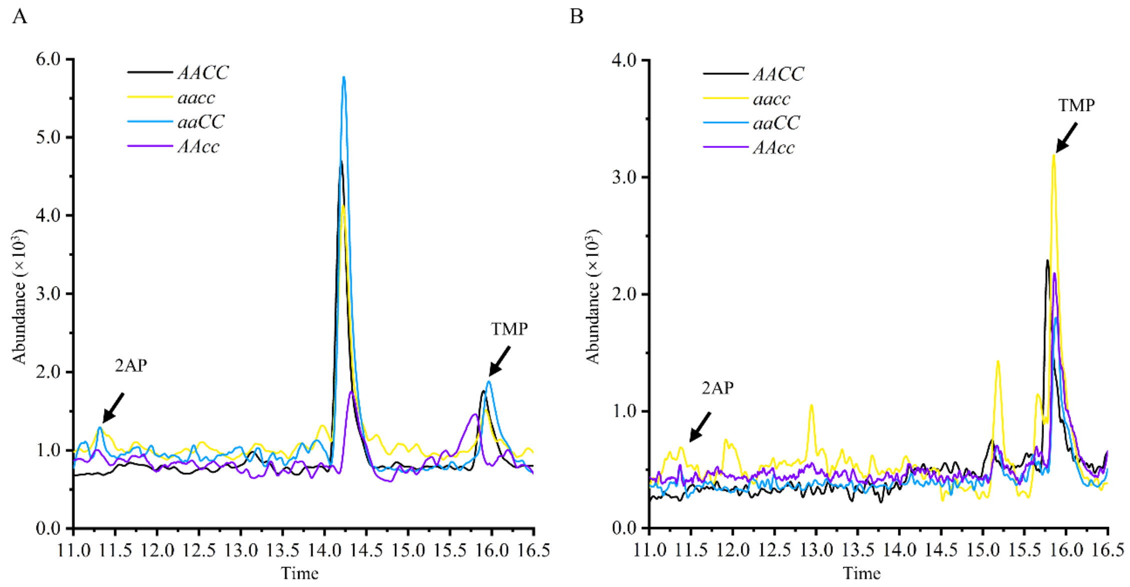

Supplementary Figure S4. Total ion chromatograms of 2-acetyl-1-pyrroline (2AP) and 2,4,6-trimethyl pyridine (TMP, as an internal standard) in flowers and seeds of the *Bnaaldh10a9* mutants and wild type (J9712). AACC, J9712; aacc, *bnaa06.aldh10a9 bnac03.aldh10a9* double mutant; aaCC, *bnaa06.aldh10a9* single mutant; AAcc, *bnac03.aldh10a9* single mutant. (A) flowers; (B) seeds.

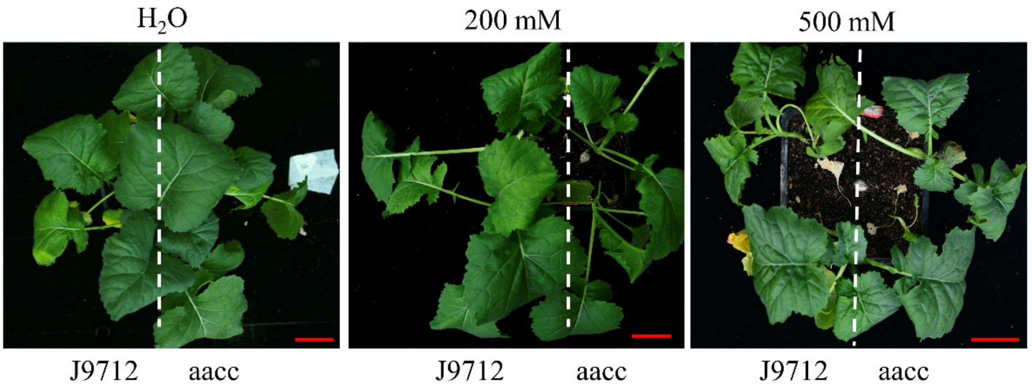

Supplementary Figure S5. Salt stress tolerance of the *bnaa06.aldh10a9 bnac03.aldh10a9* double mutant (aacc) and wild type. Plants were grown on nutrient soil for 20 d, then treated with H_2O, 200 mM NaCl and 500 mM NaCl and grown for another 8 d before being photographed. Experiments were carried out for three independent biological replicates. Bar = 5 cm.

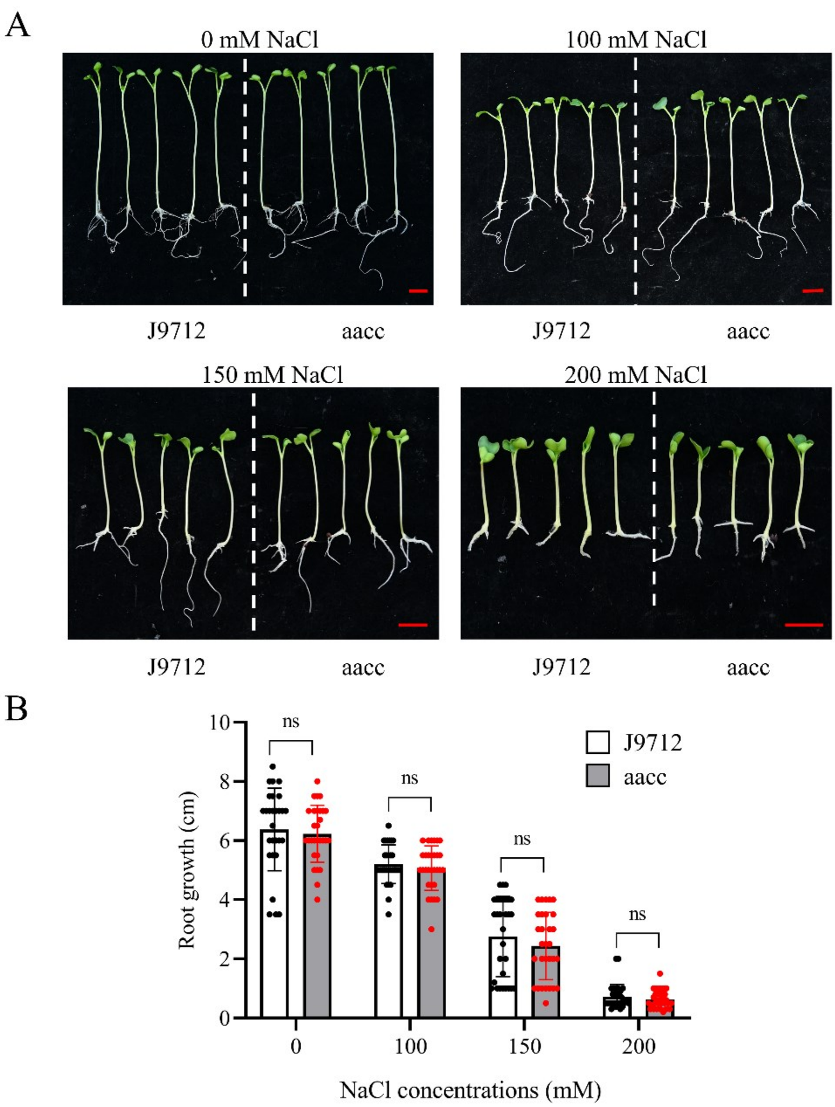

Supplementary Figure S6. Root growth of *bnaa06.aldh10a9 bnac03.aldh10a9* double mutant (aacc) and wild type (J9712) under varying salt concentrations. (A) Seeds were sown and germinated on half strength MS medium for one day and then transferred to the same medium containing 0-200 mM NaCl for an additional six days before being photographed. Bar = 1 cm. (B) Quantification of primary root length on half strength MS medium containing levels of salt stress. Data are shown as the mean ± SD (n = 30). Statistical significance was determined by a two-tailed Student's t-test. ns indicates non-significant differences ($p > 0.05$).

Supplementary materials and methods

1. Plant material and growth conditions

The oilseed rape (*Brassica napus* L.) inbred line J9712, kindly provided by Prof. Yongming Zhou of Huazhong Agricultural University, served as the transformation receptor. All plants were grown in a climate chamber under a 18 h light /6 h dark photoperiod at 22 °C with a humidity of 65%.

2. Subcellular localization

The coding sequence of *BnaA06.ALDH10A9* was inserted into the pBI221-GFP vector (at the *Sac*I restriction enzyme site) with GFP at the N-terminus via homologous recombination using a ClonExpress II One Step Cloning Kit (Vazyme, China), producing the GFP-BnaA06.ALDH10A9 fusion construct.

The coding sequence of BnaA06.ALDH10A9 was inserted into the pBI221-GFP vector at the N-terminus using homologous recombination with a ClonExpress II One Step Cloning Kit (Vazyme, China) at the SacI restriction enzyme site, resulting in the GFP-BnaA06.ALDH10A9 fusion construct.

Primers used are listed in Supplementary Table S1. mCherry fused to the peroxisome localization signal serine-lysine-leucine (mCherry-SKL) was used as a peroxisome marker. The fusion constructs were then transformed into *Arabidopsis thaliana* protoplasts following the previously described protocol (Yoo et al., 2007). Protoplasts were isolated from leaf tissues of 3- to 4-week-old *Arabidopsis* plants grown on soil. Florescence in the transformed protoplasts was detected by confocal laser scanning microscope (Leica TCS SP8, Germany) with excitation at 488 nm and emission at 510 to 550 nm for GFP, and excitation at 587 nm and emission at 607 to 650 nm for mCherry.

3. CRISPR/Cas9 vector construction, plant transformation and mutant identification

Specific sgRNAs targeting *BnaALDH10A9* were designed using CRISPR-P v2.0 (Liu et al., 2017). The synthesized sgRNA oligos (Supplementary Table S1) were annealed to form double chains, and then inserted into the psgR-Cas9 (n) vector with *Bsa*I digestion (Mao et al., 2013). The recombinant CRISPR/Cas9 plasmid was

transformed into J9712 by the *Agrobacterium tumefaciens*-mediated hypocotyl method (Dai et al., 2020). Positive transgenic plants were identified by PCR with specific primers. Then, the target genomic regions were amplified by PCR with gene-specific primers in the T_0 generation. Subsequently, mutants were detected by TA cloning followed by Sanger sequencing. Primer sequences are shown in Supplementary Table S1. The homozygous and heterozygous mutations in T_1 and T_2 generations were detected using allele-specific PCR markers for *BnaA06.ALDH10A9* and *BnaC03.ALDH10A9*. The forward primers were allele-specific, distinguishing between the mutant and wild type alleles, while the reverse primers are copy-specific, distinguishing between *BnaA06.ALDH10A9* and *BnaC03.ALDH10A9* (Supplementary Figure S2). To ensure accuracy, some samples were randomly selected for verification through Sanger sequencing.

4. 2-acetyl-1-pyrroline (2AP) measurements

2AP was measured according to Chen et al. (2024) with slight modifications. Leaves from 6-week-old plants, flowers and dried mature seeds were collected from *Bnaaldh10a9* mutants and wild type J9712 plants with three biological replicates each. For the extraction of 2AP, 20 g of leaves (cut into small pieces), 10 g of flowers (similarly minced), and 5 g of seeds (ground into fine pieces) were utilized. Each sample was mixed with 150 mL of deionized water in a round-bottomed flask, which was placed on one side of a simultaneous disintegration extractor and heated to 150°C. Concurrently, in another flask, 30 mL of dichloromethane was added along with 2,4,6-trimethyl pyridine (TMP, Sigma – Aldrich, St. Louis, MO, USA) as an internal standard at a final concentration of 0.05 mg/L, and this mixture was heated to 85 °C. Following a 40 min cyclic distillation, the dichloromethane extract was collected at the extraction port. It was then dried over anhydrous sodium sulfate, filtered through a 0.22 µm sterile filter membrane, and finally transferred into a sample vial for subsequent analysis.

2AP was quantified by gas chromatography-mass spectrometry (Trace ISQ, Thermo Fisher, USA). The chromatographic conditions were described as following: the temperature program of the gas chromatography oven was kept at 40 °C for 1 min, then increased to 65 °C at a rate of 2 °C min^{-1} and held steady at this temperature for 4

min, then ramped up to 250 °C at 10 °C min^{-1}, with a flow of 1.0 mL min^{-1} of high purity helium gas as a carrier. The mass spectrometry conditions were as follows: The El ion source temperature was set to 250 °C, the scanning mode was SIM, with mass-to-charge ratios of 43, 111 and 79, 121.

2AP content was calculated using the formula: C2 = (A2 × C1 × V)/(A1 × W), where C1 is the concentration of TMP; V is the injection volume; A1 and A2 are the peak areas of TMP and 2AP respectively and W is the mass of the sample. In addition, An authentic standard of 2AP (MACKLIN, Shanghai, China) was used to confirm the gas chromatography retention time (Supplementary Figure S3).

References

Chen Y, Hua X, Li S, Zhao J, Yu H, Wang D, Yang J, Liu L. (2024) Aromatic compound 2-acetyl-1-pyrroline coordinates nitrogen assimilation and methane mitigation in fragrant rice. Curr Biol **34:** 1-10

Dai C, Li Y, Li L, Du Z, Lin S, Tian X, Li S, Yang B, Yao W, Wang J, et al. (2020) An efficient *Agrobacterium*-mediated transformation method using hypocotyl as explants for *Brassica napus*. Mol Breeding **40:** 96

Liu H, Ding Y, Zhou Y, Jin W, Xie K, Chen L-L (2017) CRISPR-P 2.0: An improved CRISPR-Cas9 tool for genome editing in plants. Mol Plant **10:** 530-532

Mao Y, Zhang H, Xu N, Zhang B, Gou F, Zhu J-K (2013) Application of the CRISPR-Cas system for efficient genome engineering in plants. Mol Plant **6:** 2008-2011

Yoo SD, Cho YH, Sheen J (2007) *Arabidopsis* mesophyll protoplasts: a versatile cell system for transient gene expression analysis. Nat Protoc **2:** 1565-1572
